# T Lymphocyte Myosin IIA is Required for Maturation of the Immunological Synapse

**DOI:** 10.3389/fimmu.2012.00230

**Published:** 2012-08-17

**Authors:** Sudha Kumari, Santosha Vardhana, Michael Cammer, Silvia Curado, Luis Santos, Michael P. Sheetz, Michael L. Dustin

**Affiliations:** ^1^Helen and Martin Kimmel Center for Biology and Medicine, Skirball Institute of Biomolecular Medicine, New York University School of MedicineNew York, NY, USA; ^2^Department of Biological Sciences, Columbia UniversityNew York, NY, USA

**Keywords:** synapse, antigen, calcium, receptors, phosphorylation, cytoskeleton, signaling

## Abstract

The role of non-muscle myosin IIA (heavy chain encoded by the *non-muscle myosin heavy chain 9* gene, *Myh9*) in immunological synapse formation is controversial. We have addressed the role of myosin IIA heavy chain protein (MYH9) in mouse T cells responding to MHC-peptide complexes and ICAM-1 in supported planar bilayers – a model for immunological synapse maturation. We found that reduction of MYH9 expression levels using *Myh9* siRNA in proliferating mouse CD4^+^ AND T cell receptor (TCR) transgenic T cells resulted in increased spreading area, failure to assemble the central and peripheral supramolecular activation clusters (cSMAC and pSMAC), and increased motility. Surprisingly, TCR microcluster speed was reduced marginally, however TCR microclusters dissipated prior to forming a cSMAC. TCR microclusters formed in the *Myh9* siRNA-treated T cells showed reduced phosphorylation of the Src family kinase (SFK) activation loop and displayed reduced cytoplasmic calcium ion (Ca^2+^) elevation. In addition, *Myh9* siRNA-treated cells displayed reduced phosphorylation of the Cas-L substrate domain – a force-dependent SFK substrate – which was observed in control siRNA-treated cells in foci throughout the immunological synapse except the cSMAC. Cas-L exhibited TCR ligation-dependent induction of phosphorylation. These results provide further evidence that T cell activation is modulated by intrinsic force-generating systems and can be viewed as a mechanically responsive process influenced by MYH9.

## Introduction

T cells are highly migratory cells that cycle through the blood, lymph, and peripheral lymphoid organs, scanning antigen presenting cells (APCs) in search of their cognate antigen (Miller et al., 2004). T cells demonstrate both remarkable fidelity and sensitivity such that they are able to recognize as few as 10 activating peptide-MHC (pMHC) complexes among approximately a million self-pMHC on the surface of an APC (Irvine et al., 2002). Recognition of activating pMHC complexes results in acute T cell arrest and stable signal integration via formation of an immunological synapse (Dustin et al., 1998; Monks et al., 1998; Grakoui et al., 1999). Signal transduction through the stable, integrin dependent immunological synapses is critical for generation of immunological memory (Scholer et al., 2008). T cells initiate signaling within seconds of contacting an APC, but the immunological synapse undergoes a maturation process over a period of a few minutes to form three supramolecular activation clusters (SMACs; Monks et al., 1998; Freiberg et al., 2002). The first to form is the distal SMAC (dSMAC), which emerges as an actin-rich ring of close contact as the T cell spreads on the antigen positive surface (Grakoui et al., 1999; Dustin and Cooper, 2000; Bunnell et al., 2001). Segregated T cell receptor (TCR) and leukocyte function-associated antigen-1 (LFA-1) microclusters form as the F-actin ring expands to initiate TCR signaling (Grakoui et al., 1999; Krummel et al., 2000; Bunnell et al., 2002; Campi et al., 2005; Yokosuka et al., 2005; Kaizuka et al., 2007). Once the dSMAC is fully extended, distal microclusters undergo centripetal transport to assemble the peripheral SMAC (pSMAC) through which larger TCR microclusters with robust signaling percolate toward the center (Yokosuka et al., 2005; Varma et al., 2006). In the last stage of synapse maturation the large TCR microclusters reach the center and undergo a transformation from individual microclusters into a stable central SMAC (cSMAC) that lacks active signaling in a mechanism dependent upon the endosomal complexes required for transport (ESCRT) component TSG101 (Varma et al., 2006; Vardhana et al., 2010). A mature synapse has all three elements, the dSMAC, pSMAC, and cSMAC. In a multifocal synapse formed between T cells and dendritic cells there are multiple cSMAC like structures (Tseng et al., 2008). The role of cytoskeletal transport in this process is well established (Mossman et al., 2005; Varma et al., 2006; DeMond et al., 2008; Hashimoto-Tane et al., 2011), but the role of the actin based motor myosin IIA (MYH9, encoded by the gene *Myh9*) has been controversial.

F-actin plays a critical role in TCR signaling and immunological synapse maturation (Valitutti et al., 1995; Varma et al., 2006). The role of MYH9 has been less clear. In pMHC specific mouse effector T cells, MYH9 is the only myosin II expressed. Initial efforts to understand the role of this myosin applied *Myh9* siRNA and inhibitors like blebbistatin. These studies suggested that MHY9 is not required for immunological synapse formation in a cell–cell system, but did not enable more detailed analysis of how these junctions formed (Jacobelli et al., 2004). In contrast, a recent study using blebbistatin with primary mouse T cells interacting with supported planar bilayers containing specific pMHC and ICAM-1 demonstrated several defects in immunological synapse formation including slowing of early actin flow and TCR microcluster translocation (Yu et al., 2012). This is consistent with earlier observations of dSMAC contractile oscillations, which are a signature of periodic non-muscle myosin II activation in lamellipodia (Dobereiner et al., 2006; Sims et al., 2007). This force-sensing signature suggested that there might be a mechanical component to TCR signaling (Sims et al., 2007). Studies with primary human T cells and the Jurkat T cell line demonstrated a profound effect of MYH9 inhibition by blebbistatin or silencing by *Myh9* siRNA on signaling and immune synapse maturation (Ilani et al., 2009; Babich et al., 2012; Yi et al., 2012). Specifically, superantigen induced activation of Jurkat T cell line by the Raji B cell line was inhibited by blebbistatin as was the interaction of the cells. Analysis of immunological synapse dynamics using ICAM-1 and anti-CD3 presented in a mobile form on planar bilayers revealed slowing or stasis of TCR microclusters and defective signaling following blebbistatin or *Myh9* siRNA treatment. The centripetal actin flow was only completely abrogated when both actin polymerization and myosin II based contractility were blocked (Yi et al., 2012). Similar results have been obtained in a system where anti-CD3 is adsorbed to glass substrates, leading to early activation of centripetal F-actin flow (Babich et al., 2012). Specific defects in TCR signaling have been variably observed, but there is a consensus that Ca^2+^ signaling is attenuated when MYH9 activity is blocked (Ilani et al., 2009; Babich et al., 2012; Yi et al., 2012; Yu et al., 2012).

The role of MYH9 in the maturation and stability of LFA-1/ICAM-1 interactions within the pSMAC is less studied, although a critical role for non-muscle myosin II in the maturation of nascent integrin-rich adhesions in other cell types has been conclusively demonstrated (Choi et al., 2008). MYH9 associates with LFA-1 upon ligation and is involved in the turnover of LFA-1–ICAM-1 interactions during migration *in vitro* (Morin et al., 2008). MYH9 immunoreactivity is highly enriched in the pSMAC and blebbistatin treatment results in defects in pSMAC formation (Yi et al., 2012; Yu et al., 2012). Overall, immunological synapse stability has not been examined.

Mechanotransdution is the process by which physical forces, such as the pushing force of actin polymerization and pulling forces of non-muscle myosin II against F-actin attached to adhesion sites, is converted into a chemical signal (Vogel and Sheetz, 2006). One of the best-characterized force sensors in non-muscle cells is the family of proteins containing a Crk associated substrate (Cas) substrate domain. In stromal cells, p130Cas has been shown to undergo phosphorylation by SFK Fyn in response to stretching of the purified protein or the protein within a live cell (Sawada et al., 2006). Phosphorylation of the Cas substrate domain in p130 Cas generates a binding site for Crk, which binds the Rap1 exchange factor C3G. Since this process takes place in the context of adhesion formation, the generation of Rap1 could be seen as a feed-forward process. The hematopoietic family member of the CAS family is Cas-L (gene *Nedd9*), which has an established role in T cell trafficking, but not activation (Regelmann et al., 2006). Treatment of T cells with the myosin II inhibitor blebbistatin or the myosin light chain kinase inhibitor ML-7 both reduce Cas-L phosphorylation in the immunological synapse (Yu et al., 2012). However, in these studies it was not determined if Cas-L phosphorylation was related to TCR signaling or purely to LFA-1 interaction with ICAM-1, which is observed in the absence of specific pMHC to activate TCR signaling. Cas-L may be of interest both functionally and as an endogenous force sensor in T cells.

In order to further clarify the role of MYH9 in immunological synapse formation with natural pMHC ligands in supported planar bilayers we have used high-resolution microscopy in combination with siRNA-mediated suppression of *MyH9*. Using siRNA targeting multiple sequences in the *Myh9* transcript, we have determined that MYH9 plays critical roles in the formation of the pSMAC and the cSMAC. Furthermore, we have found that Cas-L displays strong MYH9-dependent phosphorylation in foci throughout the immunological synapse, in response to TCR triggering. These studies support a model in which MYH9 plays a major role in immunological synapse formation, function in signaling, and stability.

## Materials and Methods

### Reagents

Affinity purified polyclonal antibodies against phospho-Src (Cell Signaling Technology – cat#6943) and Cas substrate domain (Cell Signaling Technology – cat#4015) were obtained from Cell Signaling Technology and relevant fluorescently labeled secondary antibodies were obtained from Jackson Immunoresearch. H57 Fab were obtained from Biolegend and were labeled with Alexa568. All fluorescent dyes were purchased from Molecular Probes (Invitrogen) or Amersham Biosciences and conjugated with desired proteins according to manufacturer’s protocol.

### Cell culture

Transgenic AND B10Br Mouse splenocytes were expanded in presence of 1 μM moth cytochrome C (MCC 88–103) peptide to generate AND T cell blasts. These blasts were further expanded in IL-2 (50 U/ml) containing DMEM culture media. Work with animals was carried out under the supervision of and with approval from the NYU School of Medicine Institutional Animal Care and Use Committee.

### siRNA treatment

**Figure 1:**
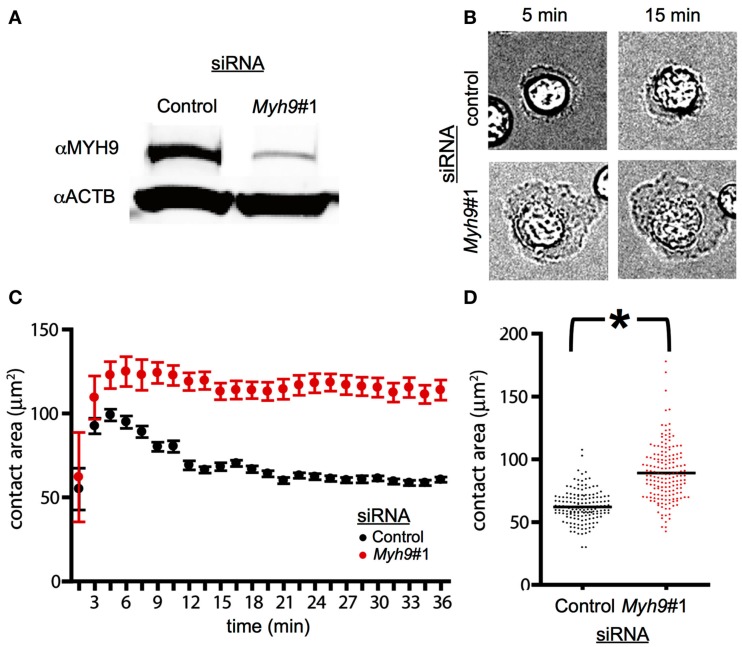
**MYH9 regulates the size of the immunological synapse**. **(A)** AND CD4+ T cells were electroporated with a control siRNA or *Myh9*#1 siRNA. The total cellular proteins were resolved by SDS-PAGE and the levels of actin and MYH9 were determined by immunoblotting to be reduced by 84%. **(B)** Bright field images of AND T cells treated with control siRNA and *Myh9*#1 siRNA on bilayer presenting ICAM-1 and I-E^k^-MCC at the indicated times. *Myh9*#1 siRNA-treated cells display larger lamellipodia at all times. **(C)** Graph representing the contact area of cells treated with control siRNA (black) and *Myh9*#1 siRNA (red) determined using IRM images at the indicated time points. Each point shows mean and standard deviation in a single field followed over 36 min. While the control cells display a distinct contraction, this is absent in *Myh9*#1 siRNA-treated cells. **(D)** Scatter plot of contact area for control (black) or *Myh9*#1 siRNA-treated cells for several fields at 30 min. *p < 0.0001. This increased contact area in *Myh9*#1 siRNA-treated T cells has been observed in 6 of 6 experiments.

**Figure 2:**
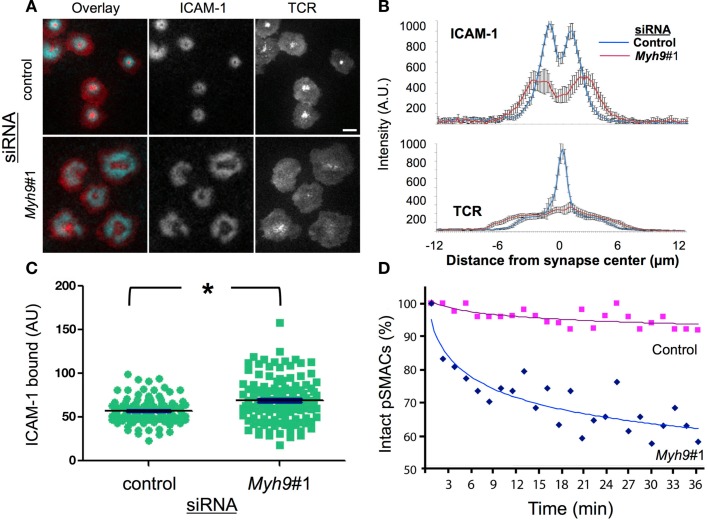
**MYH9 is required for cSMAC and pSMAC formation**. **(A)** Control and *Myh9*#1 siRNA-treated T cells on bilayers with ICAM-1 and I-E^k^-MCC after 3 min. Blue = ICAM-1 and Red = H57 Fab (TCR). Scale bar = 5 μm. **(B)** ICAM-1 (top) and TCR (bottom) population average of line scans from 14 control and 10 *Myh9*#1 siRNA-treated T cell synapses. **(C)** ICAM-1 accumulation in the immunological synapse of cells treated with control (green circle) or *Myh9*#1 (green square) siRNA at 30 min *p < 0.001. **(D)** Time-dependent symmetry breaking of ICAM-1 accumulation in control (red squares) and *Myh9*#1 (red) (blue diamonds) siRNA-treated T cells. This experiment was repeated twice.

**Figure 3:**
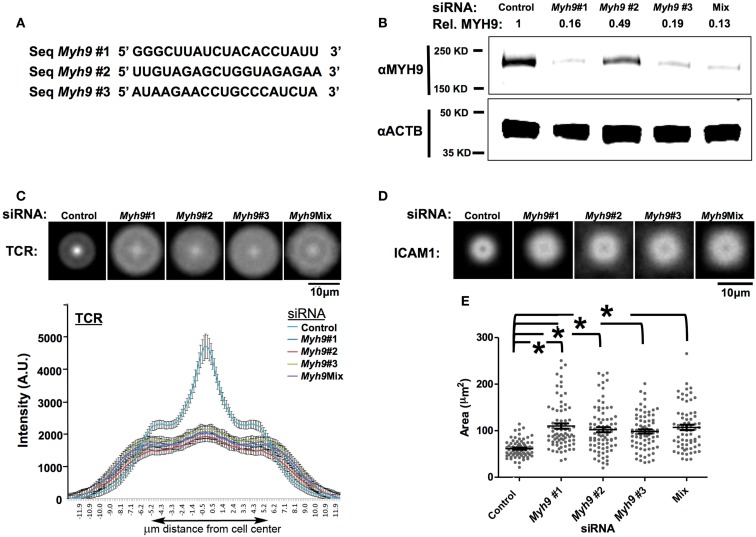
**SMAC defect in T cells is specific to MYH9 depletion**. **(A)** Sequences of three siRNA duplexes (#1, #2, #3) targeting different regions of the *Myh9* mRNA. **(B)** Western blot analysis for MYH9 expression in AND T cell blasts electroporated with the three individual *Myh9* siRNAs (#1, #2, #3), the mixture of all three siRNAs (Mix) or control siRNA. MYH9 and β-actin protein levels for each condition are shown. The value shown at the top of each lane (Rel. MYH9) represents the MYH9 band intensity normalized to actin intensity in the corresponding lane, as a proportion of the value in the control lane. **(C,D)** Each image in the top row is an average of 60 or more siRNA-treated cells (control, #1, #2, #3, Mix) registered at the centroid for Alexa568-H57 staining of TCR **(C)** or Cy5-ICAM-1 **(D)**. The plot in **(C)** shows diameter profiles of TCR (Alexa568-H57 staining) distributions with standard error of the mean through the centroid in >60 cells for each siRNA transfection. **(E)** T cell-bilayer contact area measured using interference reflection microcopy images. Each point on the plot represents the value for a single cell, and the bars represent mean values ± SEM. The mean values of contact areas are significantly higher in *Myh9* siRNA-treated cells (#1–110.1, #2–102.0, #3–98.4, Mix-107.0 μm^2^) compared to control (61.47 μm^2^). *p-values <0.0001 vs. control.

**Figure 4:**
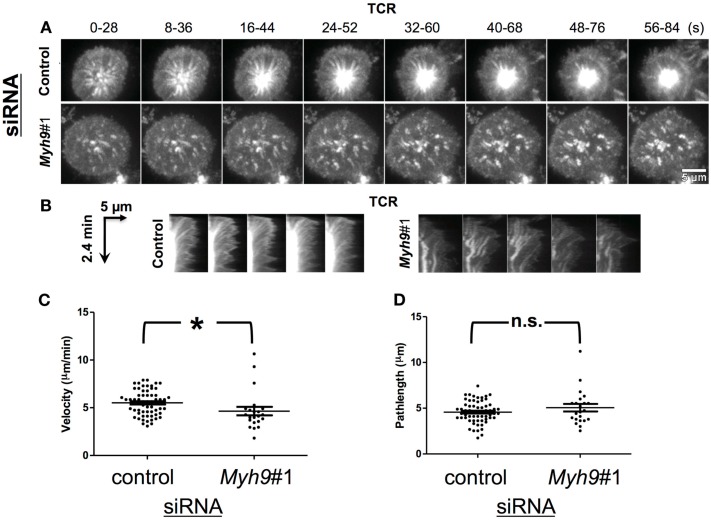
**MYH9 is not required for microcluster transport**. **(A)** Maximum intensity projection of TCR microclusters of *Myh9*#1 and control siRNA-treated cells labeled with Alexa568-H57 Fab (TCR); 30 s of imaging (15 frames) were condensed into one image every 8 s, to represent the movement of TCR microclusters. **(B)** Radial kymograms shows microcluster tracks over a period of 2.4 min in a control siRNA- or *Myh9*#1 siRNA-treated cell (arrow shows increasing time). Note that tracks in *Myh9*#1 siRNA cells are wavy and lack termination into cSMAC. **(C)** Scatter plot of TCR microcluster velocities in control and *Myh9*#1 siRNA-treated cells. Each data point represents one microcluster, the bars represent mean ± SEM. **(D)** Scatter plot showing path lengths of microclusters in control or *Myh9*#1 siRNA-treated cells. Each microcluster was manually tracked from the time of appearance till time of arrest. Each point denotes the path length value for one microcluster. The bars represent mean ± SEM. The time-lapse experiment was done thrice with similar results.

**Figure 5:**
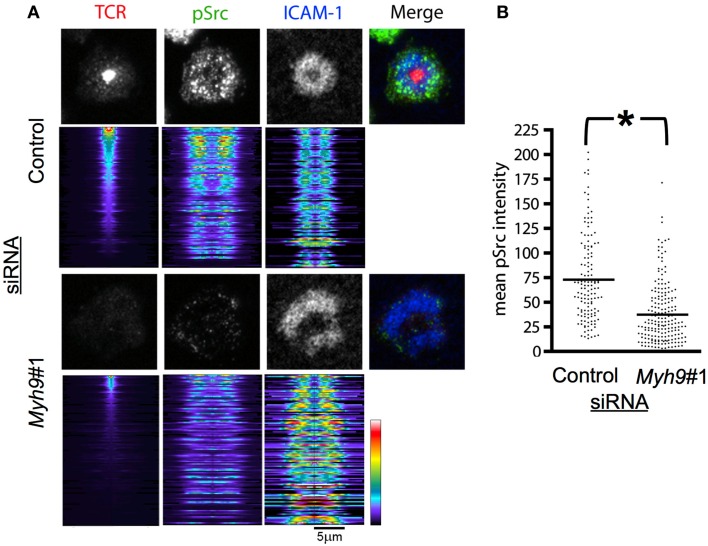
**MYH9 promotes recruitment of active SFK to TCR microclusters**. **(A)** Indirect immunofluorescence for pSrc (green), ICAM-1 (blue), and H57 Fab (TCR, red) in AND T cells treated with control or *Myh9*#1 siRNA and incubated with reconstituted bilayers for 15 min. Below each representative cell fluorescent image is the stack of radial line scans originating at the TCR centroid and sorted by highest concentration TCR cSMAC, at the top, to the lowest concentration, at the bottom. Each horizontal line represents one pseudo-colored radial average for an individual cell. *Myh9*#1 siRNA-treated cells showed reduced SFK phosphorylation. **(B)**. Scatter plot showing the mean *p*Src intensity for *Myh9*#1 and control siRNA-treated cells. The integrated *p*Src values for the contact areas were significantly different *p < 0.0001. The experiment was repeated twice with similar results.

**Figure 6:**
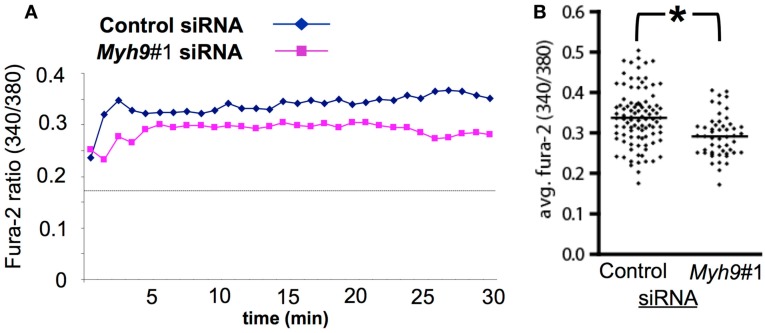
**MYH9 promotes Ca^2+^ mobilization in response to pMHC**. AND T cells were subjected to electroporation with control siRNA or *Myh9*#1 siRNA and labeled with Fura-2 immediately before imaging to determine the 340 nm /380 nm ratio, which is proportional to cytoplasmic free Ca^2+^ concentration. **(A)** Traces for control siRNA-treated cells and for *Myh9*#1 siRNA-treated cells. Each trace represents Fura-2 ratio for 93 (control) or 65 (*Myh9*#1) cells. The dashed line represents the average Fura-2 ratio for AND T cells on bilayers with ICAM-1 alone. **(B)** The average (integrated) Fura-2 ratio from 0 to 30 min with one data point for each cell. Under these conditions of labeling and imaging the average cellular baseline of Fura-2 on bilayers with ICAM-1 alone is 0.17 and the maximal ratio in the presence of ionomycin and 2 mM extracellular Ca^2+^ is 1.2. *Myh9#1 siRNA reduced the average Ca^2+^ by 27% (*p* < 0.0001). The experiment was repeated twice with the same result.

**Figure 7:**
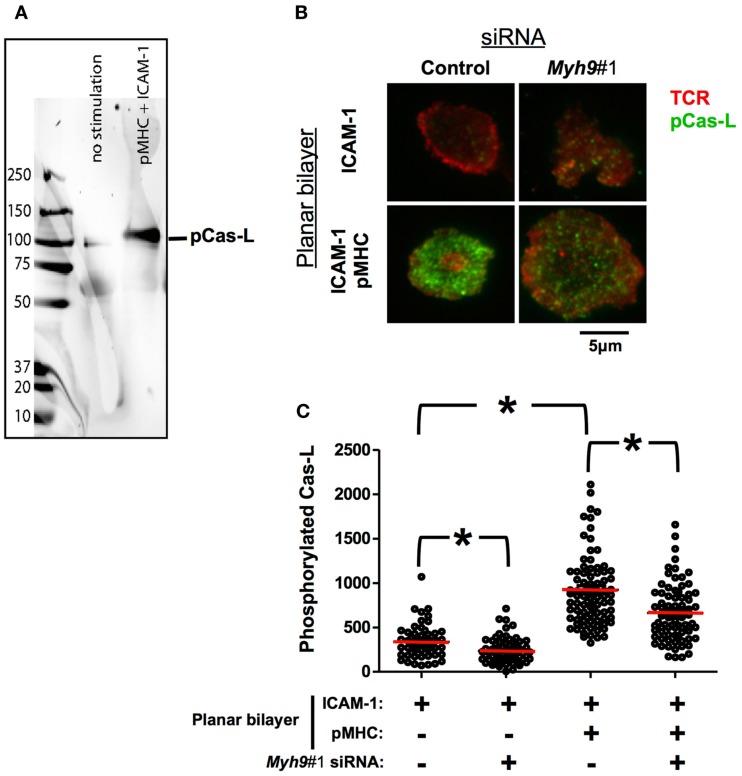
**Phosphorylation of Cas-L in the immunological synapse is MYH9-dependent**. **(A)** Western blot of non-stimulated or pMHC-ICAM-1 stimulated AND cells using an antibody against the phosphorylated Cas substrate domain-specific antibody (Y165) that specifically recognizes a 105 kDa band corresponding to p-Cas-L. **(B)** Indirect p-Cas-L immunofluorescence in control and *Myh9*#1 siRNA-treated cells incubated with reconstituted lipid bilayers containing ICAM-1 alone or ICAM-1 with 10 molecules/μm^2^ I-Ek-MCC for 3 min, imaged using TIRF microscopy. H57 Fab signal (TCR, in Pseudo-colored red) and phosphorylated Cas-L signal (in Pseudo-colored green). Both H57 and phosphorylated Cas-L signals have been contrasted to identical levels in all of the presented images. **(C)** Scatter plot of average intensity of phosphorylated Cas-L signal in TIRFM field, each point represents the value from one cell. Addition of I-E^k^-MCC to the ICAM-1 containing bilayers increased Cas-L phosphorylation 2.8-fold (*p* < 0.0001, Mann–Whitney *U*-test) In *Myh9*#1 siRNA-treated cells phosphorylated Cas-L (pCas-L) is reduced to 70% compared to control siRNA-treated cells (*p* < 0.0001, Mann–Whitney *U*-test). The red bars show the mean intensity. The experiment was repeated three times with similar results.

siRNAs targeted against mouse *Myh9* were obtained from Dharmacon: *Myh9* siRNA#1 J-040013-05; *Myh9* siRNA#2 J-040013-06; *Myh9* siRNA#3 J-040013-07. *Myh9* siRNA#1 was used unless otherwise indicated and the *Myh9* siRNA mix contains all three siRNAs (#1, #2, #3). The control non-specific siRNA was also obtained from Dharmacom: D-001810-10-20. AND T cell blasts were electroporated on day 4 with 2–4 μg of RNA duplexes using an Amaxa Nucleoporator^®^ according to manufacturer’s instructions. The amount of MYH9 protein was determined by immunoblotting on three million cells with separation on SDS-PAGE and normalization with β-actin. Proteins were detected with a Licor Odyssey scanner. 60–85% reduction of MYH9 levels was achieved with one round of electroporation (as in Figures 3, 4, and 7), whereas a 90–95% reduction could be achieved with a second round of electroporation on day 6 (as in Figures 1, 2, 5, and 6). AND T cells were utilized for experiments 2–3 days after the last siRNA treatment (Vardhana et al., 2010). After electroporation it is important to use low speed centrifugation to avoid damaging the cells as specified by the Amaxa protocol.

### Planar bilayers

Supported planar bilayers were formed in parallel plate flow cells (Bioptechs). I-E^k^-MCC88-103-6His_2_ (10–100 molecules/μm^2^) and Cy5-ICAM-1-12His (200 molecules/μm^2^) were purified from supernatants of S2 insect cells by immunoaffinity chromatography (Dustin et al., 2007). Planar lipid bilayers were formed from small unilamellar vesicles containing 12% 1,2-dioleoyl-*sn*-glycero-3-[(*N*-(5-amino-1-carboxypentyl) iminodiacetic acid) succinyl] (nickel salt; Avanti Polar Lipids; Dustin et al., 2007).

### Ca^2+^ imaging

Control and *MyH9* siRNA-treated cells were loaded with Fura-2 for 20 min at 37°C and were incubated with bilayers for 30 min (Vardhana et al., 2010). Images were acquired at a frame rate of 1 frame/min and at wavelength 340 nm (increases when Ca^2+^ is bound) and 380 nm (decreases when Ca^2+^ is bound). A background corrected ratio of 340 nm/380 nm was plotted over time as a measure of relative intracellular Ca^2+^ concentration.

### Imaging and analysis

AND T cell blasts were incubated with bilayers reconstituted with ICAM-1 and pMHC for the indicated time points and were imaged live at 37°C or were fixed using 2% paraformaldehyde (PFA) and processed for immunofluorescence. TIRF imaging was carried out using an Olympus IX70 microscope with a 60× NA 1.45 objective and a Hamamatsu Orca ER camera or a Nikon Eclipse Ti with 100× NA 1.49 objective and an Andor DU897 back illuminated EMCCD camera. Solid-state lasers (Coherent) provided illumination at 488, 561, and 641 nm and narrow pass filters (Chroma Technology) were used for detection. Illumination for Ca^2+^ imaging was provided by a 150 W Xe lamp and was performed by wide-field on an Olympus IX70 with Ludl filter wheels. Acquisition settings were maintained constant throughout each imaging procedure and between samples. Image analysis was performed with Metamorph and ImageJ. Briefly, to measure intensities, images were subtracted for background, then cells in the subtracted images were marked with ROIs, and the intensity values obtained from the ROIs were plotted as raw values in scatter plots, unless otherwise indicated. The graphs and statistical analyses were performed with Microsoft Excel and Graphpad Prism, and *p*-values were calculated using Student’s two-tailed unpaired *t*-test or Mann–Whitney *U*-test if data were not normally distributed. The experiments were performed at least twice.

## Results

### MYH9 constrains immunological synapse size

To assess the role of MYH9 in immunological synapse stability and signaling we targeted the *Myh9* mRNA by siRNA. *Myh9* encodes the heavy chain of the type II myosin in mouse T cells (Jacobelli et al., 2004). Electroporation of primary effector T cells from AND TCR transgenic mice with *Myh9*-specific siRNA#1 (*Myh9*#1) resulted in a 84% reduction in MYH9 expression as compared to control siRNA-treated T cells (Figure 1A). To evaluate immune synapse formation in a uniform manner for both control and *Myh9*#1 siRNA-treated cells we utilized supported planar bilayers containing ICAM-1 and I-E^k^-MCC. Both of the proteins have poly His tags and bind to the bilayers via Ni^2+^ chelating lipids. Bright field imaging suggested greater spreading in the *Myh9*#1 siRNA-treated T cells compared to control siRNA-treated cells (Figure 1B). To investigate SMAC formation, ICAM-1 was labeled with Cy5 and the TCR was visualized with Alexa568-H57 Fab. We tested the control siRNA and *Myh9*#1 siRNA-treated T cells in separate flow cells and allowed the cells to interact for >30 min at 37°C while capturing images every 90 s. In this first set of experiments we measured the contact area by interference reflection microscopy and tracked TCR microclusters by TIRFM. In a representative time-course from one field, the control siRNA-treated cells (*n* = 8) spread to an average maximum contact area of 100 μm^2^ (by 3 min) and then contracted to 70 μm^2^ (by 12 min). TCR microclusters formed and moved centripetally to generate a cSMAC (by 15 min; Figure 1C). In contrast, *Myh9* siRNA-treated cells (*n* = 3) spread to an average maximum contact area of 130 μm^2^ (by 6 min) and shrunk gradually over the following 30 min (Figure 1C). In a larger survey of synapse area at 30 min, the average contact area was 62.1 μm^2^ for control siRNA (*n* = 153) and 89.7 μm^2^ for *Myh9* siRNA-treated AND T cells (*n* = 170), which is significant at *p* < 0.0001 (Figure 1D). These results are consistent with earlier work on the role of non-muscle myosin II in control of stromal cell spreading (Cai et al., 2010).

### MYH9 is required for SMAC formation on planar bilayers

**Figure A1:**
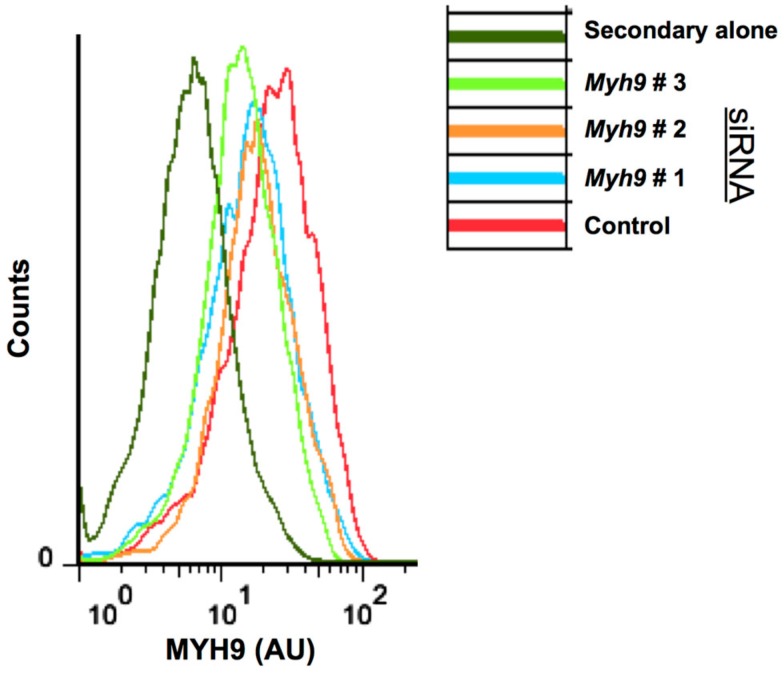
**Flow cytometric analysis of AND T cell blasts electroporated with *Myh9* siRNA**. Cells electroporated with siRNA were fixed, immunostained for MYH9 protein, and analyzed using flow cytometry. siRNA #1, #2, #3 denotes different *Myh9* siRNA sequences, shown in Figure 3A.

**Figure A2:**
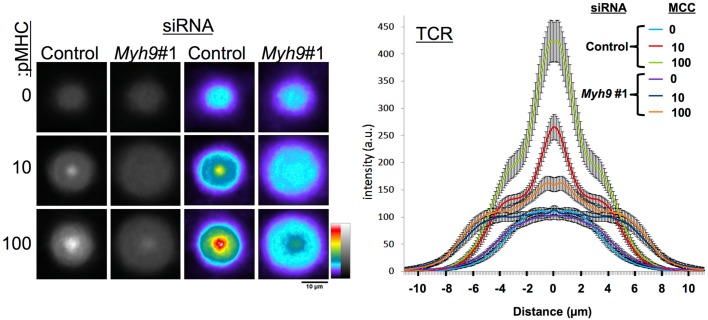
**TCR accumulation as a function of concentrations of I-E^k^-MCC at 0, 10, and 100 molecules/μm^2^ in the substrate bilayer with 200 molecules/μm^2^ ICAM-1**. Left panel shows averages of 60 or more cells treated with *Myh9*#1 or control siRNA registered at their centroids on bilayers of different protein concentrations. The graph on the right shows diameter profile plots of protein distributions with SEM across the cells from periphery to center back out through periphery.

We next investigated the role of MYH9 in the formation of the pSMAC and cSMAC by imaging Cy5-ICAM-1 in the bilayer and prelabeling TCR with Alexa568-H57 Fab, respectively, in synapses of T cells treated with a control or *Myh9*#1 siRNA (Figure 2A). H57 Fab does not impair activation of T cells by the planar bilayer (Johnson et al., 2000). In control siRNA-treated cells ICAM-1 forms a pSMAC ring and the TCR forms a cSMAC within 3 min (Figure 2A). In contrast, *Myh9*#1 siRNA impaired the consolidation of the pSMAC and cSMAC (Figure 2A). To quantify this effect we performed averaging of the radial ICAM-1 and TCR intensity for 14 control siRNA-treated and 10 *Myh9*#1 siRNA-treated T cells at 3 min. This analysis demonstrated that the control siRNA-treated cells had a pSMAC with a bimodal ICAM-1 distribution with a peak to peak diameter of 2.3 μm and a cSMAC with a unimodal H57 distribution with a width at half height of 1.5 μm (Figure 2B). *Myh9*#1 siRNA resulted in a significant broadening of the bimodal ICAM-1 distribution with an increase in the peak to peak distance to 4.4 μm and a nearly flat distribution of H57 fluorescence with a width at half height of 11 μm (Figure 2B). However, the integrated intensities of the ICAM-1 and TCR signal in the interface were 21% higher (Figure 2C) and 8% lower (not shown), respectively, in the *Myh9*#1 siRNA-treated T cells compared to the control siRNA-treated T cells. Increasing the time of observation from 3 to 30 min did not change the distribution of TCR or ICAM-1 and revealed that the *Myh9* siRNA-treated AND T cells broke their nascent immunological synapses approximately eight times faster than the control siRNA-treated AND T cells (Figure 2D, Movie S1 in Supplementary Material). Thus, we conclude that MYH9 is required to form the pSMAC and cSMAC based on quantitative analysis of protein distribution and immunological synapse stability. To control for potential non-specific effects of *Myh9*#1 siRNA, we tested two additional siRNAs targeting different regions of the *Myh9* transcript (Figure 3A). When assessed for MYH9 protein silencing capability, all three siRNA sequences alone (#1, #2, or #3) or in combination (Mix) displayed consistent reduction in MYH9 levels (Figure 3B, Figure A1 in Appendix). Cells treated with these siRNAs alone (#1, #2, or #3) or in combination (Mix) exhibited comparable defects among each other in cSMAC (Figure 3C – averaged images and line scan) and pSMAC (Figure 3D – average images only) contraction and an increase in cell contact area (Figure 3E), compared to cells transfected with control siRNA. These results indicate that defects in cSMAC and pSMAC observed upon MYH9 expression reduction of 60% or greater are likely to be due to depletion of MYH9 protein specifically, rather than an off target effect, which would be different for each siRNA sequence. To further address whether the involvement of MYH9 in synapse maturation is dependent on pMHC density, and perhaps could be overcome by high pMHC dose, we monitored cSMAC formation on bilayers reconstituted with varying pMHC concentrations while keeping ICAM-1 density constant. We examined cSMAC intensity in the absence of pMHC or in the presence of 10 or 100 molecules of I-E^k^-MCC per μm^2^. At the highest pMHC density tested (100 molecules of I-E^k^-MCC per μm^2^) a weak cSMAC (central TCR accumulation) in cells treated with *Myh9*#1 siRNA was observed but was still significantly attenuated compared to the control siRNA experiment (Figure A2 in Appendix). This result suggests that the role of MYH9 cannot be overcome by higher pMHC concentration.

### MYH9 is not required for TCR microcluster transport

TCR signal transduction is mediated by formation of TCR microclusters that are generated in the dSMAC and traverse the pSMAC. TCR microclusters have been reported to move at 1.5–5.6 μm/min (Yokosuka et al., 2005; Varma et al., 2006). We analyzed TCR microcluster movement after synapse formation based on imaging movies of H57 Fab-tagged TCR acquired at one frame every 2 s using maximum intensity projection over 28 s [28 s of imaging (15 frames) were condensed into one image every 8 s; Figure 4A] as well as radial kymographs or kymographs from individually tracked microcluster paths (Figure 4B). The microcluster speed was determined from the slope of the line formed by the microcluster given that the abscissa is time(s) and the ordinate is distance (μm; Figure 4B). We made measurements on 62 microclusters in control siRNA-treated T cells and 22 microclusters in *Myh9*#1 siRNA-treated T cells. In contrast to the MYH9 dependence of anti-CD3 induced microcluster transport reported previously (Ilani et al., 2009), transport of pMHC induced TCR microclusters was only marginally dependent on MYH9 (Figure 4C, *p* < 0.01). The average centripetal velocity was 5.51 ± 1.32 μm/min in control siRNA-treated cells and 4.64 ± 2.0 μm/min in *Myh9*#1 siRNA-treated cells. The TCR microclusters exhibited comparable average path length (4.6 μm for control siRNA; 5.0 μm for *Myh9*#1 siRNA) before merging into cSMAC (control siRNA) or track termination (*Myh9*#1 siRNA; Figure 4D, *p* = 0.57, Mann–Whitney *U*-test). Furthermore, the tracks in *Myh9*#1 siRNA-treated cells displayed more meandering compared to directed movement in control cells (Figure 4B), as previously reported in the Jurkat model (Yi et al., 2012). Thus, the failure of MHY9-depleted cells to form a cSMAC can be attributed to the failure of synapse contraction and therefore a greater distance for the microclusters to traverse as well as the reduced directional movement.

### MYH9 increases Src family kinase activation

Recruitment of activated SFK to TCR microclusters is one of the earliest steps in T cell activation (Nika et al., 2010). T cell signaling is sustained in the synapse by continually forming new microclusters such that accumulation of active SFK at TCR microclusters will be seen at all times during T cell activation (Campi et al., 2005; Varma et al., 2006). We examined SFK activation loop phosphorylation (pSrc) in microclusters in AND T cells interacting with bilayers containing pMHC and ICAM-1 for 15 min, staining the T cells with an antibody against the phosphorylated SFK activation loop, which is highly conserved between Lck and Fyn, the major SFKs of T cells, and imaging by TIRFM to focus on the signal in the immunological synapse. We analyzed the images by generating radial intensity profile plots (Figure 5A). The radial sweeps originate at the centroid of the TCR signal and are stratified by cSMAC intensity. Control siRNA-treated cells displayed efficient cSMAC and pSMAC formation and activation loop phosphorylation throughout the pSMAC and dSMAC (Figure 5A). *Myh9*#1 siRNA-treated T cells displayed a dramatically reduced cSMAC and disorganized peripheral accumulation of both ICAM-1 and the phosphorylated SFK activation loop (Figure 5A). Overall, the level of active SFK recruited to the synapse decreased by 48.7% in *Myh9*#1 siRNA-treated compared to control siRNA-treated T cells (Figure 5B, *p* < 0.0001).

### MYH9 contributes to TCR-dependent cytoplasmic Ca^2+^ elevation

We next determined if Ca^2+^ elevation was impaired in *Myh9* siRNA-treated cells using Fura-2 ratiometric imaging in live cells incubated on supported planar bilayers with ICAM-1 and agonist pMHC at 2 molecules/μm^2^. Control siRNA-treated T cells displayed a significant increase in the 340 nm/380 nm ratio indicating elevated cytoplasmic Ca^2+^ relative to cells interacting with ICAM-1 alone (Figure 6A, dashed line), as expected (Varma et al., 2006). We found that *Myh9*#1 siRNA-treated T cells showed significantly impaired antigen-induced Ca^2+^ elevation (Figure 6A). *Myh9*#1 siRNA-treated cells displayed a blunted initial spike in Ca^2+^ (Figure 6A) and a 27% decrease in integrated Ca^2+^ elevation over 30 min, compared to control siRNA-treated cells (Figure 6B, *p* < 0.0001). These results suggest that MYH9 contributes significantly to cytoplasmic Ca^2+^ elevation that is an integral component in T cell activation.

### Phosphorylation of Cas-L substrate domain is MYH9-dependent

It has been suggested that the TCR might act as a mechanotransducer that is triggered in part by mechanical forces that could be applied externally or generated by the T cell (Sims et al., 2007; Kim et al., 2009; Li et al., 2010). To test the hypothesis that T cells generate sufficient force to activate a bona fide mechanotransduction system we assessed the phosphoshorylation of the Cas substrate domain of Cas-L. The Cas protein family has been shown to be activated by mechanical unfolding and subsequence phosphorylation of the substrate domain (Sawada et al., 2006). Lymphocytes express one member of the Cas family called Cas-L, which has an established role in lymphocyte migration (Regelmann et al., 2006). It has been recently reported that Cas-L is phosphorylated in the immunological synapse, it was not determined if this phosphorylation is dependent upon TCR activation (Yu et al., 2012). As Cas-L is not required for TCR signaling, we utilized it here as a reporter of forces in the immunological synapse. As expected, we detected a single band of 105 kDa in activated mouse T cells using the phospho-Cas substrate domain antibody (Figure 7A).

Control siRNA-treated AND T cells activated on ICAM-1 and I-E^k^-MCC showed a significantly higher Cas-L phosphorylation (924 ± 46 U) than on ICAM-1 only bilayers (331 ± 26 U) as observed by TIRF imaging (Figures 7B,C; *p* < 0.0001). However, a decrease in Cas-L phosphorylation in cells treated with *Myh9* siRNA was observed both in ICAM-1 only (235 ± 17 U; Figures 7B,C; *p* < 0.0005 vs. control siRNA) and on ICAM-1 and I-E^k^-MCC bilayers (661 ± 37 U; Figures 7B,C; *p* < 0.0001 vs. control siRNA), compared to control siRNA-treated cells. Thus, the decreased phosphorylation of the Cas substrate domain in cells treated with *Myh*9 siRNA suggests that there is significant MYH9-dependent force generation in the immunological synapse, but also a significant MYH9 independent component.

## Discussion

There has been significant recent interest in the hypothesis that TCR triggering involves mechanotransduction, the conversion of mechanical signals into chemical signals. This concept was first advanced at the molecular level and later extended to incorporate cytoskeletal forces into models. The critical relationship of antigen receptors to actomyosin cytoskeleton was first noted in the 1970s and the role of actin polymerization in TCR signaling was most clearly illustrated in 1995 (Braun et al., 1978; Valitutti et al., 1995). In addition to the TCR, T lymphocytes also utilize integrin family adhesion molecules to increase sensitivity to antigenic pMHC and for effector function. Integrins are the best-studied receptor systems for mechanotransduction involving cytoskeletal linkage. In this study we extend early evidence that MHY9 contributes to T cell activation. We have used *Myh9* siRNA as the major tool to perturb MYH9 function. We confirmed the specificity of this effect by assessing major phenotypes using three independent *Myh9* siRNAs. Our studies extend earlier results with antigenic complexes and superantigens in cell–cell systems (Jacobelli et al., 2004; Ilani et al., 2009) and anti-CD3 and pMHC presented by supported bilayers (Ilani et al., 2009; Yi et al., 2012; Yu et al., 2012). We concur with earlier studies that immune synapse formation is disrupted in the presence of reduced levels of MYH9 (Ilani et al., 2009; Yi et al., 2012; Yu et al., 2012), and we have uncovered a number of features that were not previously explored. We found that treatment with *Myh9* siRNA increased the area of T cell synapses, decreased the stability of T cell synapses, increased LFA-1–ICAM-1 interaction, decreased accumulation of TCR microclusters at the cSMAC, decreased SFK activation at TCR microclusters, decreased peak and integrated cytoplasmic Ca^2+^ elevation, and decreased pMHC-dependent phosphorylation of Cas-L.

We found that all three *Myh9* siRNAs tested increased the area of contact between T cells and supported planar bilayers presenting MHC-peptide complexes. This paradoxical result is consistent with results in stromal cells that also demonstrated excessive spreading under several conditions in which MYH9 levels were reduced or inhibited (Cai et al., 2010). This effect has been described in terms of MYH9 providing a cytoplasmic “coherence” that prevents fragmentation of the contact into dendritic shapes as protrusion and spreading continues (Cai et al., 2010). A practical aspect of this effect for T cells is that MYH9 allows lamellae to bridge adhesion sites over gaps of several microns (Rossier et al., 2010), which may be useful for migration in complex 3D settings like lymph nodes. Importantly, T cells were unable to assemble a cSMAC and pSMAC structure when subjected to any of the three *Myh9* siRNAs. This result is consistent with a number of recent studies that reveal defects in microcluster transport, signaling, and pSMAC formation in cells treated with blebbistatin or *Myh9* siRNA. We previously found that human T cells treated with blebbistatin or *Myh9* siRNA display profound inhibition of anti-CD3 triggered TCR microclusters and a decrease in signaling triggered by superantigen mediated activation by APCs (Ilani et al., 2009). Blebbistatin reduced TCR microcluster velocity and increased meandering in Jurkat cells stimulated with anti-CD3 (Yi et al., 2012). Another study using the same TCR transgenic system and agonist MHC-peptide complex as our current study also found that blebbistatin or myosin light chain kinase inhibitors particularly inhibited the early rapid phase of TCR microcluster transport (Yu et al., 2012). An earlier study did not report defects in immunological synapse formation in antigen specific T cell-APCs conjugates, but did detect defects in T cell polarity and motility (Jacobelli et al., 2004). Conditional knock-out of *Myh9* in T cells allowed further dissection of the motility defects and concurs with our results on increased spreading (Jacobelli et al., 2009). Loss of *Myh9* in human T cells has also been associated with defects in de-adhesion from ICAM-1 (Morin et al., 2008). Our results in which *Myh9* siRNA-treated T cells become more motile on substrates with ICAM-1 and agonist MHC-peptide complexes may reflect the loss of coherence in the cytoskeleton noted by Cai et al. (2010). The observation that LFA-1–ICAM-1 interactions increased may be due to reduced forces acting on the interactions allowing for a longer duration of interaction (Huppa et al., 2010). While lymphocytes do not undergo the fragmentation of the contact area observed in stromal cells in the absence of myosin II activity, we did observe symmetry breaking and motility in *Myh9*#1 siRNA-treated T cells under conditions where control siRNA-treated T cells displayed mostly stable, symmetric immunological synapses. Thus, the key structure that defines the stable immunological synapse, the pSMAC, appears to be dependent on cytoplasmic coherence generated by MYH9.

Our examination of signaling in microclusters focused on recruitment of active SFKs. Ilani et al. (2009) found that anti-CD3 antibodies presented with ICAM-1 on planar bilayers generated microclusters with normal levels of SFK activation, but decreased ZAP-70 and LAT signaling in the presence of blebbistatin or *MyH9* siRNA. More recently, studies with mouse T cells similar to those performed here confirmed defects in ZAP-70 phosphorylation in TCR microclusters (Yu et al., 2012). With agonist pMHC engaging both TCR and CD4, we found that *Myh9* siRNA reduced SFK activation in TCR microclusters. The discrepancy with our earlier work in Ilani et al. may reflect the role of CD4 in recruiting SFK and may also implicate CD4 in mechanotransduction. It is important to point out that current biochemical analysis suggests that SFKs like Lck are constitutively active in T cells and triggering likely depends upon concentration of the active kinases at TCR to overcome tonic inhibition by phosphatases (Nika et al., 2010). We have also examined exclusion of the phosphatase CD45 from microclusters in the presence of blebbistatin and found that CD45 is still excluded from microclusters (data not shown). We did not directly investigate LAT recruitment and phosphorylation but observed decreased Ca^2+^ signaling in *Myh9* siRNA-treated T cells. Ca^2+^ elevation is downstream of LAT recruitment of phospholipase C-γ. Similar results were obtained using the same TCR transgenic model upon blebbistatin treatment (Yu et al., 2012). Recent studies in Jurkat T cells also found a critical role for centripetal actin flow in PLC-γ activation (Babich et al., 2012).

There appear to be at least two actin networks in motile cells: the lamellipodium, which is the leading structure rich in dynamic actin, followed by the lamella, rich in tropomyosin (Ponti et al., 2004). The lamellipodium undergoes contractile oscillations that are myosin II dependent (Giannone et al., 2004, 2007; Dobereiner et al., 2006). We have proposed that in the immunological synapse, the dSMAC is the equivalent of the lamellipodium and the pSMAC is the equivalent of the lamella (Sims et al., 2007). The lamellipodium is an important sensory structure and thus the dSMAC may have a similar role in sensing the mechanics of the T cell’s environment and APCs. T cells are sensitive to the mechanics of their environment. Mouse T cells are sensitive to substrates rigidity (Judokusumo et al., 2012). Interestingly treatment with blebbistatin decreased mechanosensing by mouse T cells (Judokusumo et al., 2012) supporting a critical role of myosin II in mechanosensing during T cell activation. Human T cells showed enhanced responses on softer substrates, suggesting applications in immunotherapy (O’Connor et al., 2012). The pSMAC environment may be particularly important for sustaining signaling and amplifying Ca^2+^ signals. A role for ICAM-1 in costimulating Ca^2+^ signals has been previously noted with no mechanism (Wulfing et al., 1998). T cells can sense single agonist pMHC (Irvine et al., 2002), but require multiple pMHC for full commitment to priming or effector function. Linking TCR triggering to forces generated by F-actin-myosin II networks may provide a convenient mechanism for coincidence detection in TCR triggering to increase fidelity of T cells activation.

Cas-L is related to p130Cas, which is phosphorylated under force-dependent unfolding of the substrate domain in cell free and intact cell systems (Sawada et al., 2006). The antibody against the Cas substrate domain is the same as for p130Cas and Cas-L (Figure 6A). Supported planar bilayers are laterally fluid and it is unlikely that the lipid linked pMHC and ICAM-1 provide much resistance to F-actin mediated pushing forces or actomyosin-mediated pulling. One possibility is that forces in the immunological synapse that are leading to pMHC-dependent increases in Cas-L phosphorylation are based on internal resistance to pushing or pulling. On the other hand, by protein prediction algorithms, the Cas substrate domain may be unstructured (R. Bonneau, unpublished observations) and the force needed to unfold this domain is too small to be measured using magnetic tweezers (M. P. S., unpublished observations), suggesting that the viscosity of the planar bilayer membrane might be sufficiently resistant to allow Cas-L substrate domain unfolding and phosphorylation. Knock-out of Cas-L results in reduced T cell motility, but with no obvious effects on T cell activation (Regelmann et al., 2006). However, Cas-L partners with SLP-76 and Pyk2 in T cells and recent findings show that Pyk2 is involved in CD8 T cell differentiation (Beinke et al., 2010). These results suggest that mechanotranduction may have roles beyond TCR triggering and motility. The phosphorylation of the Cas substrate domain leads to the recruitment of the adapter Crk (Sawada et al., 2006). Phosphorylation of CrkL has recently been implicated in negative regulation of natural killer cells (Liu et al., 2012). It remains to be determined if this pathway is relevant to positive or negative regulation of T cell activation. Cas-L may have implications for T cell mechanotransduction as the role of Cas-L in more biological contexts is explored.

## Conflict of Interest Statement

The authors declare that the research was conducted in the absence of any commercial or financial relationships that could be construed as a potential conflict of interest.

## Supplementary Material

The Supplementary Material for this article can be found online at http://www.frontiersin.org/T_Cell_Biology/10.3389/fimmu.2012.00230/abstract

Supplementary Movie S1 **Immunological synapse defects in *Myh9*#1 siRNA-treated T cells: pSMAC formation and immunological synapse stability**. Image of Cy5-ICAM-1 accumulated in AND TCR transgenic T cells treated with control siRNA (Ctrl) or *Myh9*#1 siRNA (MyH9 KD) and interacting with supported planar bilayers containing 20 molecules/μm^2^ of I-E^k^-MCC and 200 molecules/μm^2^ of Cy5-ICAM-1. The interval between frames is 1 min. Movie is related to Figure 2. Ctrl cells form a well defined pSMAC and remain in place over 24 min, whereas *Myh9*#1 siRNA cells fail to form a normal pSMAC and initiate migration.

Supplementary Movie S2 **Immunological synapse defects in *Myh9*#1 siRNA-treated T cells: cSMAC formation**. Image of Alexa568-H57 Fab labeled AND TCR transgenic T cells treated with control siRNA (Ctrl) or *Myh9*#1 siRNA and interacting with supported planar bilayers containing 20 molecules/μm^2^ of I-E^k^-MCC and 200 molecules/μm^2^ of Cy5-ICAM-1. The interval between frames is 6 s. Movie is directly related to Figure 4. Ctrl siRNA-treated TCR microclusters form and are transported to the cSMAC, whereas *Myh9*#1 siRNA-treated TCR microclusters are transported, but tend to dissipate some distance from the central location where the cSMAC would normally form.

## References

[B1] Babich A. Li S. O’Connor R. S. Milone M. C. Freedman B. D. Burkhardt J. K. (2012). F-actin polymerization and retrograde flow drive sustained PLC gamma 1 signaling during T cell activation. J. Cell Biol. 197, 775–787 10.1083/jcb.201201018 22665519 PMC3373411

[B2] Beinke S. Phee H. Clingan J. M. Schlessinger J. Matloubian M. Weiss A. (2010). Proline-rich tyrosine kinase-2 is critical for CD8 T-cell short-lived effector fate. Proc. Natl. Acad. Sci. U.S.A. 107, 16234–16239 10.1073/pnas.1011556107 20805505 PMC2941278

[B3] Braun J. Fujiwara K. Pollard T. D. Unanue E. R. (1978). Two distinct mechanisms for redistribution of lymphocyte surface macromolecules. II. Contrasting effects of local anesthetics and a calcium ionophore. J. Cell Biol. 79, 419–426 10.1083/jcb.79.2.409 363728 PMC2110236

[B4] Bunnell S. C. Hong D. I. Kardon J. R. Yamazaki T. McGlade C. J. Barr V. A. Samelson L. E. (2002). T cell receptor ligation induces the formation of dynamically regulated signaling assemblies. J. Cell Biol. 158, 1263–1275 10.1083/jcb.200203043 12356870 PMC2173229

[B5] Bunnell S. C. Kapoor V. Trible R. P. Zhang W. Samelson L. E. (2001). Dynamic actin polymerization drives T cell receptor-induced spreading: a role for the signal transduction adaptor LAT. Immunity 14, 315–329 10.1016/S1074-7613(01)00112-1 11290340

[B6] Cai Y. Rossier O. Gauthier N. C. Biais N. Fardin M. A. Zhang X. Miller L. W. Ladoux B. Cornish V. W. Sheetz M. P. (2010). Cytoskeletal coherence requires myosin-IIA contractility. J. Cell. Sci. 123, 413–423 10.1242/jcs.058297 20067993 PMC2816186

[B7] Campi G. Varma R. Dustin M. L. (2005). Actin and agonist MHC-peptide complex-dependent T cell receptor microclusters as scaffolds for signaling. J. Exp. Med. 202, 1031–1036 10.1084/jem.20051182 16216891 PMC1373686

[B8] Choi C. K. Vicente-Manzanares M. Zareno J. Whitmore L. A. Mogilner A. Horwitz A. R. (2008). Actin and alpha-actinin orchestrate the assembly and maturation of nascent adhesions in a myosin II motor-independent manner. Nat. Cell Biol. 10, 1039–1050 10.1038/ncb1763 19160484 PMC2827253

[B9] DeMond A. L. Mossman K. D. Starr T. Dustin M. L. Groves J. T. (2008). T cell receptor microcluster transport through molecular mazes reveals mechanism of translocation. Biophys. J. 94, 3286–3292 10.1529/biophysj.107.119099 18199675 PMC2275686

[B10] Dobereiner H. G. Dubin-Thaler B. J. Hofman J. M. Xenias H. S. Sims T. N. Giannone G. Dustin M. L. Wiggins C. H. Sheetz M. P. (2006). Lateral membrane waves constitute a universal dynamic pattern of motile cells. Phys. Rev. Lett. 97, 038102 10.1103/PhysRevLett.97.038102 16907546

[B11] Dustin M. L. Cooper J. A. (2000). The immunological synapse and the actin cytoskeleton: molecular hardware for T cell signaling. Nat. Immunol. 1, 23–29 10.1038/76877 10881170

[B12] Dustin M. L. Olszowy M. W. Holdorf A. D. Li J. Bromley S. Desai N. Widder P. Rosenberger F. van der Merwe P. A. Allen P. M. Shaw A. S. (1998). A novel adapter protein orchestrates receptor patterning and cytoskeletal polarity in T cell contacts. Cell 94, 667–677 10.1016/S0092-8674(00)81608-6 9741631

[B13] Dustin M. L. Starr T. Varma R. Thomas V. K. (2007). “Supported planar bilayers for study of the immunological synapse,” in Current Protocols in Immunology, eds Coligan J. E. Bierer B. E. Margulies D. H. Shevach E. M. Strober W. (New York: John Wiley and Sons, Inc.), Unit 18.13. 10.1002/0471142735.im1813s76 18432988

[B14] Freiberg B. A. Kupfer H. Maslanik W. Delli J. Kappler J. Zaller D. M. Kupfer A. (2002). Staging and resetting T cell activation in SMACs. Nat. Immunol. 3, 911–917 10.1038/ni836 12244310

[B15] Giannone G. Dubin-Thaler B. J. Dobereiner H. G. Kieffer N. Bresnick A. R. Sheetz M. P. (2004). Periodic lamellipodial contractions correlate with rearward actin waves. Cell 116, 431–443 10.1016/S0092-8674(04)00058-3 15016377

[B16] Giannone G. Dubin-Thaler B. J. Rossier O. Cai Y. Chaga O. Jiang G. Beaver W. Dobereiner H. G. Freund Y. Borisy G. Sheetz M. P. (2007). Lamellipodial actin mechanically links Myosin activity with adhesion-site formation. Cell 128, 561–575 10.1016/j.cell.2006.12.039 17289574 PMC5219974

[B17] Grakoui A. Bromley S. K. Sumen C. Davis M. M. Shaw A. S. Allen P. M. Dustin M. L. (1999). The immunological synapse: a molecular machine controlling T cell activation. Science 285, 221–227 10.1126/science.285.5425.221 10398592

[B18] Hashimoto-Tane A. Yokosuka T. Sakata-Sogawa K. Sakuma M. Ishihara C. Tokunaga M. Saito T. (2011). Dynein-driven transport of T cell receptor microclusters regulates immune synapse formation and T cell activation. Immunity 34, 919–931 10.1016/j.immuni.2011.05.012 21703543

[B19] Huppa J. B. Axmann M. Mortelmaier M. A. Lillemeier B. F. Newell E. W. Brameshuber M. Klein L. O. Schutz G. J. Davis M. M. (2010). TCR-peptide-MHC interactions in situ show accelerated kinetics and increased affinity. Nature 463, 963–967 10.1038/nature08746 20164930 PMC3273423

[B20] Ilani T. Vasiliver-Shamis G. Vardhana S. Bretscher A. Dustin M. L. (2009). T cell antigen receptor signaling and immunological synapse stability require myosin IIA. Nat. Immunol. 10, 531–539 10.1038/nrg2603 19349987 PMC2719775

[B21] Irvine D. J. Purbhoo M. A. Krogsgaard M. Davis M. M. (2002). Direct observation of ligand recognition by T cells. Nature 419, 845–849 10.1038/nature01076 12397360

[B22] Jacobelli J. Bennett F. C. Pandurangi P. Tooley A. J. Krummel M. F. (2009). Myosin-IIA and ICAM-1 regulate the interchange between two distinct modes of T cell migration. J. Immunol. 182, 2041–2050 10.4049/jimmunol.0803267 19201857

[B23] Jacobelli J. Chmura S. A. Buxton D. B. Davis M. M. Krummel M. F. (2004). A single class II myosin modulates T cell motility and stopping, but not synapse formation. Nat. Immunol. 5, 531–538 10.1038/ni1065 15064761

[B24] Johnson K. G. Bromley S. K. Dustin M. L. Thomas M. L. (2000). A supramolecular basis for CD45 tyrosine phosphatase regulation in sustained T cell activation. Proc. Natl. Acad. Sci. U.S.A. 97, 10138–10143 10.1073/pnas.97.3.958 10963676 PMC27752

[B25] Judokusumo E. Tabdanov E. Kumari S. Dustin M. L. Kam L. C. (2012). Mechanosensing in T lymphocyte activation. Biophys. J. 102, L5–L7 10.1016/j.bpj.2011.11.043 22339876 PMC3260692

[B26] Kaizuka Y. Douglass A. D. Varma R. Dustin M. L. Vale R. D. (2007). Mechanisms for segregating T cell receptor and adhesion molecules during immunological synapse formation in Jurkat T cells. Proc. Natl. Acad. Sci. U.S.A. 104, 20296–20301 10.1073/pnas.0710258105 18077330 PMC2154425

[B27] Kim S. T. Takeuchi K. Sun Z. Y. Touma M. Castro C. E. Fahmy A. Lang M. J. Wagner G. Reinherz E. L. (2009). The alphabeta T cell receptor is an anisotropic mechanosensor. J. Biol. Chem. 284, 31028–31037 10.1074/jbc.M109.000752 19755427 PMC2781503

[B28] Krummel M. F. Sjaastad M. D. Wulfing C. Davis M. M. (2000). Differential clustering of CD4 and CD3ζ during T cell recognition. Science 289, 1349–1352 10.1126/science.289.5483.1349 10958781

[B29] Li Y. C. Chen B. M. Wu P. C. Cheng T. L. Kao L. S. Tao M. H. Lieber A. Roffler S. R. (2010). Cutting Edge: mechanical forces acting on T cells immobilized via the TCR complex can trigger TCR signaling. J. Immunol. 184, 5959–5963 10.4049/jimmunol.0900775 20435924

[B30] Liu D. Peterson M. E. Long E. O. (2012). The adaptor protein Crk controls activation and inhibition of natural killer cells. Immunity 36, 600–611 10.1016/j.immuni.2012.03.007 22464172 PMC3355982

[B31] Miller M. J. Hejazi A. S. Wei S. H. Cahalan M. D. Parker I. (2004). T cell repertoire scanning is promoted by dynamic dendritic cell behavior and random T cell motility in the lymph node. Proc. Natl. Acad. Sci. U.S.A. 101, 998–1003 10.1073/pnas.0306407101 14722354 PMC327133

[B32] Monks C. R. Freiberg B. A. Kupfer H. Sciaky N. Kupfer A. (1998). Three-dimensional segregation of supramolecular activation clusters in T cells. Nature 395, 82–86 10.1038/25764 9738502

[B33] Morin N. A. Oakes P. W. Hyun Y. M. Lee D. Chin E. Y. King M. R. Springer T. A. Shimaoka M. Tang J. X. Reichner J. S. Kim M. (2008). Nonmuscle myosin heavy chain IIA mediates integrin LFA-1 de-adhesion during T lymphocyte migration. J. Exp. Med. 205, 195–205 10.1084/jem.20071543032708c 18195072 PMC2234359

[B34] Mossman K. D. Campi G. Groves J. T. Dustin M. L. (2005). Altered TCR signaling from geometrically repatterned immunological synapses. Science 310, 1191–1193 10.1126/science.1119238 16293763

[B35] Nika K. Soldani C. Salek M. Paster W. Gray A. Etzensperger R. Fugger L. Polzella P. Cerundolo V. Dushek O. Hofer T. Viola A. Acuto O. (2010). Constitutively active Lck kinase in T cells drives antigen receptor signal transduction. Immunity 32, 766–777 10.1016/j.immuni.2010.05.011 20541955 PMC2996607

[B36] O’Connor R. S. Hao X. Shen K. Bashour K. Akimova T. Hancock W. W. Kam L. C. Milone M. C. (2012). Substrate rigidity regulates human T cell activation and proliferation. J. Immunol. 189, 1330–1339 10.4049/jimmunol.1102757 22732590 PMC3401283

[B37] Ponti A. Machacek M. Gupton S. L. Waterman-Storer C. M. Danuser G. (2004). Two distinct actin networks drive the protrusion of migrating cells. Science 305, 1782–1786 10.1126/science.1100533 15375270

[B38] Regelmann A. G. Danzl N. M. Wanjalla C. Alexandropoulos K. (2006). The hematopoietic isoform of Cas-Hef1-associated signal transducer regulates chemokine-induced inside-out signaling and T cell trafficking. Immunity 25, 907–918 10.1016/j.immuni.2006.09.014 17174122

[B39] Rossier O. M. Gauthier N. Biais N. Vonnegut W. Fardin M. A. Avigan P. Heller E. R. Mathur A. Ghassemi S. Koeckert M. S. Hone J. C. Sheetz M. P. (2010). Force generated by actomyosin contraction builds bridges between adhesive contacts. EMBO J. 29, 1055–1068 10.1038/emboj.2010.2 20150894 PMC2845274

[B40] Sawada Y. Tamada M. Dubin-Thaler B. J. Cherniavskaya O. Sakai R. Tanaka S. Sheetz M. P. (2006). Force sensing by mechanical extension of the Src family kinase substrate p130Cas. Cell 127, 1015–1026 10.1016/j.cell.2006.09.044 17129785 PMC2746973

[B41] Scholer A. Hugues S. Boissonnas A. Fetler L. Amigorena S. (2008). Intercellular adhesion molecule-1-dependent stable interactions between T cells and dendritic cells determine CD8+ T cell memory. Immunity 28, 258–270 10.1016/j.immuni.2007.12.016 18275834

[B42] Sims T. N. Soos T. J. Xenias H. S. Dubin-Thaler B. Hofman J. M. Waite J. C. Cameron T. O. Thomas V. K. Varma R. Wiggins C. H. Sheetz M. P. Littman D. R. Dustin M. L. (2007). Opposing effects of PKC theta and WASp on symmetry breaking and relocation of the immunological synapse. Cell 129, 773–785 10.1016/j.cell.2007.03.037 17512410

[B43] Tseng S. Y. Waite J. C. Liu M. Vardhana S. Dustin M. L. (2008). T cell-dendritic cell immunological synapses contain TCR-dependent CD28-CD80 clusters that recruit protein kinase Ctheta. J. Immunol. 181, 4852–4863 18802089 10.4049/jimmunol.181.7.4852 PMC2556893

[B44] Valitutti S. Dessing M. Aktories K. Gallati H. Lanzavecchia A. (1995). Sustained signaling leading to T cell activation results from prolonged T cell receptor occupancy. Role of T cell actin cytoskeleton. J. Exp. Med. 181, 577–584 10.1084/jem.181.2.577 7836913 PMC2191861

[B45] Vardhana S. Choudhuri K. Varma R. Dustin M. L. (2010). Essential role of ubiquitin and TSG101 protein in formation and function of the central supramolecular activation cluster. Immunity 32, 531–540 10.1016/j.immuni.2010.04.005 20399684 PMC2905630

[B46] Varma R. Campi G. Yokosuka T. Saito T. Dustin M. L. (2006). T cell receptor-proximal signals are sustained in peripheral microclusters and terminated in the central supramolecular activation cluster. Immunity 25, 117–127 10.1016/j.immuni.2006.04.010 16860761 PMC1626533

[B47] Vogel V. Sheetz M. (2006). Local force and geometry sensing regulate cell functions. Nat. Rev. Mol. Cell Biol. 7, 265–275 10.1038/nrm1890 16607289

[B48] Wulfing C. Sjaastad M. D. Davis M. M. (1998). Visualizing the dynamics of T cell activation: intracellular adhesion molecule 1 migrates rapidly to the T cell/B cell interface and acts to sustain calcium levels. Proc. Natl. Acad. Sci. U.S.A. 95, 6302–6307 10.1073/pnas.95.11.6302 9600960 PMC27665

[B49] Yi J. Wu X. S. Crites T. Hammer J. A. III. (2012). Actin retrograde flow and actomyosin II arc contraction drive receptor cluster dynamics at the immunological synapse in Jurkat T cells. Mol. Biol. Cell 23, 834–852 10.1091/mbc.E11-08-0731 22219382 PMC3290643

[B50] Yokosuka T. Sakata-Sogawa K. Kobayashi W. Hiroshima M. Hashimoto-Tane A. Tokunaga M. Dustin M. L. Saito T. (2005). Newly generated T cell receptor microclusters initiate and sustain T cell activation by recruitment of Zap70 and SLP-76. Nat. Immunol. 6, 1253–1262 10.1038/ni1272 16273097

[B51] Yu Y. Fay N. C. Smoligovets A. A. Wu H. J. Groves J. T. (2012). Myosin IIA modulates T cell receptor transport and CasL phosphorylation during early immunological synapse formation. PLoS ONE 7, e30704 10.1371/journal.pone.0030704 22347397 PMC3275606

